# Reliability and Validity of the Japanese Version of the Multidimensional Evaluation Scale for Patient Impression Change (MPIC): A Brief Tool for Multidimensional Assessment in Interdisciplinary Pain Management

**DOI:** 10.3390/jcm14196851

**Published:** 2025-09-27

**Authors:** Morihiko Kawate, Yihuan Wu, Yuta Shinohara, Saki Takaoka, Chisato Tanaka, Shizuko Kosugi, Kenta Wakaizumi

**Affiliations:** 1 Interdisciplinary Pain Center, Keio University Hospital, Tokyo 160-8582, Japan; morihikok2@keio.jp (M.K.); yihuanwu1@hotmail.com (Y.W.); y-shinohara@keio.jp (Y.S.); saki.takaoka@keio.jp (S.T.); c.tanaka1203@keio.jp (C.T.); shizuko.kosugi@gmail.com (S.K.); 2 Department of Anesthesiology, Keio University School of Medicine, Tokyo 160-8582, Japan

**Keywords:** multidimensional evaluation scale for patient impression change (MPIC), chronic pain, reliability, validity, interdisciplinary pain management

## Abstract

**Background:** Chronic pain significantly impacts quality of life and may lead to physical and psychological dysfunction. Although various tools have been developed to assess pain-related conditions, many are composed of time-consuming multi-item questionnaires, limiting practicality in clinical settings. The Multidimensional Evaluation Scale for Patient Impression of Change (MPIC) was developed as a simple, retrospective tool to assess multiple domains targeted in interdisciplinary pain management. This study evaluated the reliability and validity of the Japanese MPIC in patients with chronic non-cancer pain. **Methods:** We recruited 101 participants from the Interdisciplinary Pain Center at Keio University Hospital between August 2022 and September 2024. Pretreatment measures included pain intensity, disability, catastrophizing, self-efficacy, psychological distress, and sleep quality. Baseline assessments encompassed pain intensity, disability, catastrophizing, self-efficacy, psychological distress, and sleep quality. Psychological distress was evaluated using the Hospital Anxiety and Depression Scale (HADS) for the initial cohort of 35 participants and the Kessler Psychological Distress Scale (K6) for the subsequent 66 participants. Following the intervention, participants completed the MPIC, in addition to reassessments of pain intensity, disability, catastrophizing, self-efficacy, psychological distress (HADS or K6), and sleep quality. Retesting the MPIC was performed in a small subgroup of 20 participants for test–retest reliability analysis. Confirmatory factor analysis (CFA), average variance extracted (AVE), Pearson’s correlations with pain-related measures, Cronbach’s alpha, and intraclass correlation coefficients (ICC) were used to assess construct validity, convergent validity, criterion validity, internal consistency, and reliability. **Results:** CFA indicated marginal fit (CFI = 0.86, RMSEA = 0.23, SRMR = 0.06), with factor loadings from 0.49 to 0.91. AVE supported convergent validity (0.58). Internal consistency was excellent (Cronbach’s alpha = 0.93), and ICC was moderate (0.52). MPIC domains correlated significantly with changes in pain intensity, disability, catastrophizing, self-efficacy, sleep, and psychological distress (*p* < 0.05), supporting criterion validity. **Conclusions:** The Japanese MPIC provides preliminary evidence of validity and reliability, with acceptable internal consistency, marginal structural fit, and moderate test–retest reliability. These findings suggest that the MPIC may serve as a concise retrospective instrument for assessing multidimensional treatment outcomes within interdisciplinary pain management programs for chronic non-cancer pain.

## 1. Introduction

Interdisciplinary pain management is a comprehensive approach that integrates various therapeutic modalities to address the multifaceted nature of chronic pain. This method recognizes that effective management of chronic pain requires not only alleviating pain sensations but also considering the psychological, social, and functional aspects of a patient’s experience. Chronic pain conditions, such as low back pain, shoulder stiffness, and joint pain, consistently rank among the top symptoms reported in the National Health Survey in Japan, underscoring the significant impact of pain on health and quality of life [1].

In addition to reducing daily activity levels, chronic pain can lead to mood disorders such as depression and anxiety, as well as sleep disturbances. These factors contribute to a cycle of suffering that may ultimately result in the transition from acute to chronic pain. Therefore, while pain relief is a critical goal of clinical pain management, it is imperative to recognize that patients may seek to attain broader objectives related to their overall well-being. Given the complexity of pain, it is essential to evaluate the multidimensional factors associated with pain comprehensively. However, traditional pain assessments often require the completion of multiple questionnaires with numerous items, which can be time-consuming and burdensome for patients already suffering from pain. Moreover, in clinical practice, healthcare providers may find it impractical to administer multiple assessments routinely, which limits their ability to deliver personalized treatment strategies tailored to individual patient needs. This highlights a substantial need for streamlined evaluation methods in interdisciplinary pain management contexts.

To facilitate effective assessment, Farrar et al. developed the Patient Global Impression of Change (PGIC) as a single-item questionnaire. Patients use a seven-point scale to evaluate their overall impression of their status, ranging from 1 (very much improved) to 7 (very much worse) [2]. The PGIC has shown a strong association with clinically meaningful changes in pain intensity in trials comparing pregabalin to placebo among pain patients [2]. Recognizing the importance of considering multiple dimensions of pain, Gagnon et al. expanded on the original PGIC by developing the Multidimensional Evaluation Scale for Patient Impression of Change (MPIC). This tool incorporates seven additional domains that reflect key goals of interdisciplinary chronic pain management, including overall health, pain, sleep, mood, physical functioning, coping strategies, management of pain flare-ups, and medication effectiveness [3]. A notable advantage of the MPIC is that it functions as a retrospective measure, eliminating the need for a pretreatment evaluation. This characteristic allows for assessment of treatment effects at the time of responding, thereby reducing the burden on both patients and healthcare providers. In our previous study, we translated the MPIC into Japanese in collaboration with academic professionals and conducted thorough linguistic validation [4]. The objective of the current study is to evaluate the reliability and validity of the Japanese version of the MPIC in clinical settings among patients experiencing non-cancer pain.

## 2. Materials and Methods

### 2.1. Study Design

A retrospective study was conducted, and all data were collected from electronic clinical records. Opportunity to opt out was provided to all participants by distributing the study information on the web, after this study was approved by the Ethics Committee, Keio University School of Medicine (approval number: 20231048) on 4 August 2023. We routinely investigate multifaceted aspects of patients with pain, including pain intensity and location, pain quality, pain-related psychology, disability, and negative emotions. We use multiple questionnaires on a tablet device for screening at the first visit to our institution. According to the attending physician’s decision, a second assessment including the MPIC is performed to evaluate the treatment effect. Because we considered the second assessment as a post-treatment assessment in this study, intervals between pre- and post-treatment spread in a wide range depending on each patient’s condition.

### 2.2. Participants

**Figure 1:**
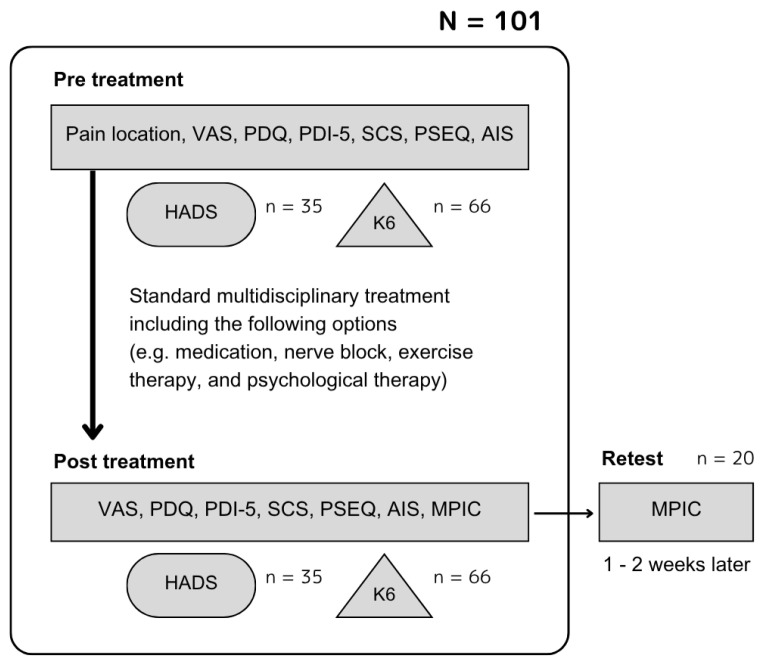
Flow diagram of study participants. VAS: Visual Analog Scale, PDQ: PainDETECT Questionnaire, PDI-5: five-item version of Pain Disability Index, SCS: Symptom Catastrophizing Scale, PSEQ: Pain Self-Efficacy Questionnaire, AIS: Athens Insomnia Scale, HADS: Hospital Anxiety and Depression Scale, K6: Kessler Psychological Distress Scale, MPIC: Multidimensional Evaluation Scale for Patient Impression Change.

Data were collected from 101 patients, aged between 20 and 85 years and native Japanese speakers, with non-cancer pain, attending the outpatient clinic at the Interdisciplinary Pain Center, Keio University Hospital, from August 2022 to September 2024. Given that a sample size of 5 to 10 times the number of questionnaire items (or at least 100 participants) is required for validity assessment, approximately 100 participants were recruited for the MPIC, which consists of eight domains, each assessed by a single item. The MPIC had been retested within 1–2 weeks after the initial MPIC assessment in 20 participants (Figure 1).

### 2.3. Measures

Demographic data (age, sex, and body mass index [BMI]) and pain duration were retrieved from electronic medical records. Pretreatment assessments included the following questionnaires: Visual Analog Scale (VAS), PainDETECT Questionnaire (PDQ), five-item version of the Pain Disability Index (PDI-5), Symptom Catastrophizing Scale (SCS), Pain Self-Efficacy Questionnaire (PSEQ), Hospital Anxiety and Depression Scale (HADS), Kessler 6-item Psychological Distress Scale (K6), and the Athens Insomnia Scale (AIS). Initially, we used HADS for psychological screening for the first 35 participants but later replaced it with K6 for the subsequent 66 participants, which comprehensively assesses stress, depression, and anxiety with concise items, making it more suitable for rapid outpatient screening as well as reducing participant burden.

Participants received standard multidisciplinary care (medication, nerve blocks, exercise therapy, and psychotherapy) between pre- and post-treatment assessments. Since post-treatment assessments were conducted at clinician-determined intervals, there were various durations between pre- and post-treatment assessments.

#### 2.3.1. Visual Analog Scale

We assessed pain intensity over a preceding week using the VAS (0–100 mm), with 0 representing “no pain” and 100 representing “the worst imaginable pain.” Studies have shown no clinically significant difference between digital and traditional paper-based VAS measurements [5].

#### 2.3.2. PainDETECT Questionnaire

The PDQ, developed by Freynhagen et al., is a screening tool for neuropathic pain, translated into Japanese by Sumitani et al. and validated by Matsubayashi et al. (Cronbach’s alpha = 0.80) [6]. The main component of the PDQ rates seven pain symptoms on a 6-point scale from 0 (never) to 5 (very strongly), with a total score of 0 to 35. A greater score indicates that there is more likely to be neuropathic pain.

#### 2.3.3. Pain Disability Index

PDI is a widely recognized self-reported measure of pain-related disability that has been extensively validated [7,8,9,10]. The original PDI consists of seven questions regarding daily living disabilities. For this study, we employed a five-item version to reduce participant burden, excluding questions related to sexual activity and life-support activities from a seven-item version of the PDI. The five-item version of the PDI, translated and validated in Japanese (Cronbach’s alpha = 0.93) [8,9], assesses how pain interferes with five domains of daily living: home, social, recreational, occupational, and self-care, using an 11-point scale from 0 (no disability) to 10 (total disability). The total score ranges from 0 to 70, with higher scores indicating greater functional disability.

#### 2.3.4. Symptom Catastrophizing Scale

The SCS evaluates catastrophic thoughts related to symptoms. It was adapted from the Pain Catastrophizing Scale (PCS) to better fit chronic mental health conditions not necessarily associated with pain. To minimize participant burden, the number of items was reduced from 13 to 7, employing a simplified response scale of 0 (never), 1 (sometimes), and 2 (often). Higher scores indicate greater catastrophizing. The original SCS has demonstrated reliability and validity in evaluating catastrophic thinking related to severe mental health conditions [11]. The Japanese version was validated through forward and back translation by academic professionals [12].

#### 2.3.5. Pain Self-Efficacy Questionnaire

The PSEQ assesses confidence in achieving desired outcomes in daily and social activities despite pain [13]. This questionnaire consists of 10 items; each rated from 0 (not confident at all) to 6 (completely confident). Total score ranges from 0 to 60, with higher scores indicating greater confidence in managing pain. The Japanese version has demonstrated strong reliability and validity (Cronbach’s alpha = 0.94, intraclass correlation coefficient = 0.80) [14].

#### 2.3.6. Psychological Measures

Participants completed either the Japanese version of the Kessler Psychological Distress Scale-6 items (K6) or the Hospital Anxiety and Depression Scale (HADS). The HADS identifies anxiety and depression in non-psychiatric hospital patients [15,16]. It comprises two subscales, anxiety (HADS-A) and depression (HADS-D), each with seven items rated on a 4-point scale. Higher scores suggest more severe psychological distress, with both subscales exhibiting robust validation (Cronbach’s alpha: HADS-A = 0.83; HADS-D = 0.82) [17].

The K6, a well-validated scale (Cronbach’s alpha = 0.85) [18], includes six items that assess feelings of nervousness and worthlessness over the past 30 days. Participants rated their distress from 0 (never) to 4 (always), with higher scores indicating greater psychological distress; scores ≥ 13 indicate severe distress.

Although the HADS and K6 differ in item content and scoring methodologies, both scales assess psychological distress and are conceptually comparable. Prior research has demonstrated a strong correlation between the two measures (r = 0.84, *p* < 0.001), supporting their interchangeability [19].

#### 2.3.7. Athens Insomnia Scale

AIS is a reliable self-administered psychometric tool (Cronbach’s alpha = 0.88) [20] used to evaluate sleep disorders. It consists of eight items rated from 0 (no problem) to 3 (severe problem), with total scores ranging from 0 to 24. Higher scores indicate more severe insomnia. Poor sleep quality and insufficient sleep duration are associated with a higher risk of chronic pain [21].

#### 2.3.8. Multidimensional Evaluation Scale for Patient Impression of Change

The Multidimensional Evaluation Scale for Patient Impression of Change (MPIC) is based on the Patient Global Impression of Change (PGIC), originally developed by Farrar et al. [2]. The PGIC assesses patients’ impression regarding perceived overall change since the initiation of treatment. Gagnon et al. expanded the PGIC to include seven additional domains: overall, pain, sleep, mood, physical functioning, cope with pain, manage pain flare-ups, and medication effectiveness, aligning with the goals of the interdisciplinary pain treatment [3]. Participants rated each domain from 1 (very much improved) to 7 (very much worse), with 4 (no change).

The English version of the MPIC has good reliability with Cronbach’s alpha = 0.89 [3]. The Japanese version was validated through a formal process involving both forward and back translation executed by academic professionals, and its comprehensibility was further confirmed with a small group of volunteers [4].

### 2.4. Data Analysis

Paired *t*-tests were performed to compare pre- and post-treatment scores of pain-related measures. Prior to the CFA, an exploratory factor analysis (EFA) was conducted to examine the underlying structure of the MPIC. The number of factors was determined using the criterion of the eigenvalue greater than 1.0. Confirmatory factor analysis (CFA) was conducted to estimate the factor loadings for the hypothesized one-factor model of the MPIC, as proposed in prior studies [3]. Goodness-of-fit was assessed using several indices, specifically the root mean square error of approximation (RMSEA), comparative fit index (CFI), and standardized root mean square residual (SRMR). An RMSEA value of <0.05 indicates a good fit, while a value of ≥0.1 suggests a poor fit [22]. A CFI of ≥0.90 indicates good fit; values between 0.80 and 0.90 suggest marginal fit, whereas values ≤ 0.80 suggest poor fit [23]. An SRMR value of ≤0.08 also indicates good fit [24].

To evaluate construct validity, the average variance extracted (AVE) was calculated, with an acceptance criterion of ≥0.5 [25]. Reliability analysis was carried out using Cronbach’s alpha to assess internal consistency; a Cronbach’s alpha coefficient greater than 0.7 suggests good internal consistency [26,27]. Test–retest reliability was analyzed using the intraclass correlation coefficient (ICC) for participants who completed the MPIC retest. Scores of each domain at post-treatment and retest were compared by Pearson’s correlation analysis. ICC values ≤ 0.5 indicate poor reliability, values from 0.5 to 0.75 suggest moderate reliability, values from 0.75 to 0.9 indicate good reliability, and values > 0.90 reflect excellent reliability [28].

Finally, Pearson’s correlation coefficients were computed between the eight domains of the MPIC and changes in measures (VAS, PDQ, PDI-5, SCS, PSEQ, AIS, HADS-A, HADS-D, and K6) to assess criterion-related validity. The HADS and K6 were analyzed separately in the present study.

Statistical analyses were implemented using JMP^®^ version 17.0.0 (SAS Institute, Cary, NC, USA) software and IBM SPSS Statistics^®^ version 30 (IBM. Armonk, NY, USA), with significance levels set at a two-tailed *p*-value < 0.05, or a false discovery rate-corrected *p*-value < 0.05 for multiple comparisons. Test–retest reliability was evaluated using the intraclass correlation coefficient (ICC) based on a two-way random-effects model with absolute agreement and single measures.

## 3. Results

### 3.1. Patient Demographics

Table 1 presents the demographic characteristics of the 101 participants. The average age was 54.5 years, and 66% of participants were female. The median duration of treatment was 169 days, with the shortest duration being 28 days. Table 2 presents treatment outcome measures. Significant improvements were observed in the VAS, PDQ, PDI, SCS, PSEQ, HADS-A, K6, and AIS compared to pretreatment. The averaged scores across all MPIC domains were below 4, specifically: overall, 3.3; pain, 3.4; sleep, 3.7; mood, 3.4; physical functioning, 3.7; cope with pain, 3.3; manage pain flare-ups, 3.4; and medication effectiveness, 3.4.

### 3.2. Reliability and Validity of MPIC

**Figure 2:**
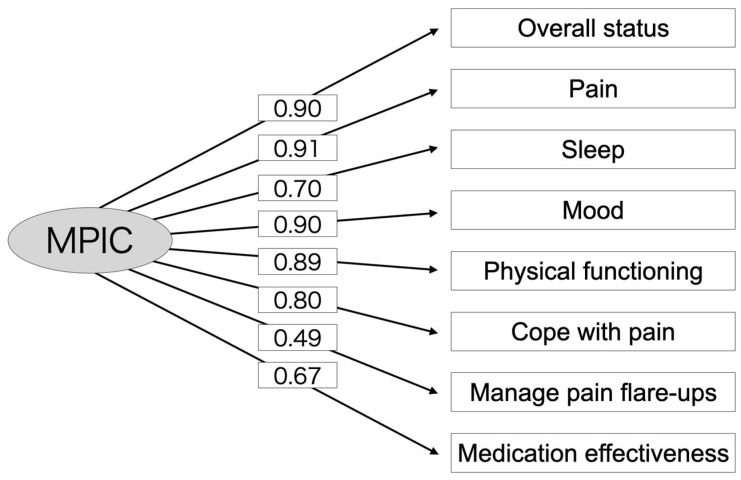
Confirmatory factor analysis for eight domains of the MPIC. Numbers represent factor loadings for each domain. MPIC: Multidimensional Evaluation Scale for Patient Impression of Change.

**Figure 3:**
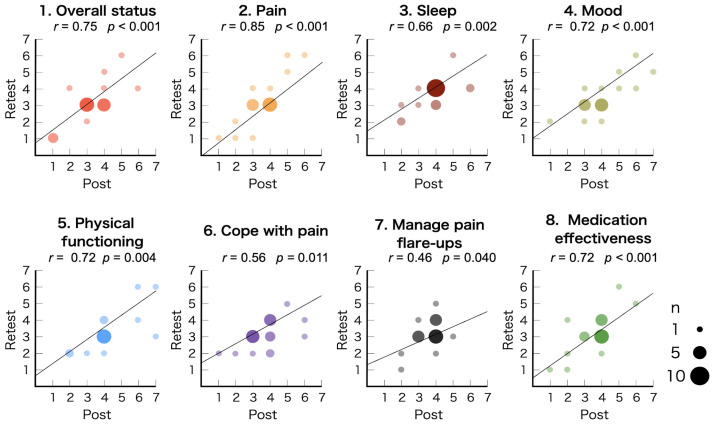
Correlation of the MPIC scores between post-treatment and retest (n = 20). The size of each circle represents the number of participants. Pearson’s correlation coefficient was calculated to assess the association between post and retest scores. MPIC: Multidimensional Evaluation Scale for Patient Impression of Change. The horizontal axis represents MPIC scores at post-treatment, and the vertical axis represents MPIC scores retested 1–2 weeks after post-treatment.

EFA indicated a one-factor solution with an eigenvalue exceeding 1.0, explaining 67% of the variance. This finding was consistent with results reported by Gagnon et al. [3]. The results of the CFA are illustrated in Figure 2, displaying the standardized factor loadings for each MPIC domain: overall, 0.90; pain, 0.91; sleep, 0.70; mood, 0.90; physical functioning, 0.89; cope with pain, 0.80; manage pain flare-ups, 0.49; and medication effectiveness, 0.67. Table 3 summarizes the validity and reliability analyses along with their acceptance criteria: CFI, 0.86; RMSEA, 0.23; SRMR, 0.06; AVE, 0.58; Cronbach’s alpha, 0.93; and ICC, 0.52 (two-way random effect model, 95% CI 0.37–0.71). All MPIC domains showed significant Pearson’s correlation coefficient between scores at post-treatment and retest (Figure 3): overall, 0.75 (*p* < 0.001); pain, 0.85 (*p* < 0.001); sleep, 0.66 (*p* = 0.002); mood, 0.72 (*p* < 0.001); physical functioning, 0.72 (*p* = 0.004); cope with pain, 0.56 (*p* = 0.011); manage pain flare-ups, 0.46 (*p* = 0.040); and medication effectiveness, 0.72 (*p* < 0.001).

### 3.3. Correlations Between MPIC Domains and Pain-Related Measures

**Figure 4:**
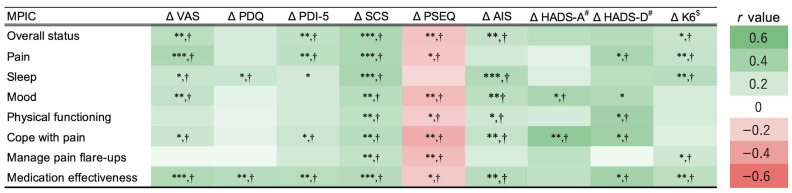
Heatmap of Pearson’s correlation coefficients between changes in pain-related measures and scores of each MPIC domain. ^#^ HADS and ^$^ K6 were analyzed in 35 and 66 participants, respectively. MPIC: Multidimensional Evaluation Scale for Patient Impression Change, VAS: Visual Analog Scale, PDQ: PainDETECT Questionnaire, PDI-5: five-item version of Pain Disability Index, SCS: Symptom Catastrophizing Scale, PSEQ: Pain Self-Efficacy Questionnaire, HADS-A: Hospital Anxiety and Depression Scale—anxiety, HADS-D: Hospital Anxiety and Depression Scale—depression, K6: Kessler Psychological Distress Scale, AIS: Athens Insomnia Scale. * *p* < 0.05, ** *p* < 0.01, *** *p* < 0.001, † false discovery rate-corrected *p* < 0.05.

Statistically significant correlations were observed between overall status and changes in VAS, PDI-5, SCS, PSEQ, AIS, and K6 (Figure 4). The pain domain correlated with VAS, PDI-5, SCS, AIS, HADS-D, and K6. Sleep correlated with VAS, PDQ, SCS, AIS, and K6. Mood correlated with VAS, SCS, PSEQ, AIS, and HADS-A. Physical functioning correlated with SCS, PSEQ, AIS, and HADS-D. Cope with pain correlated with all pain-related measures except PDQ and K6. Manage pain flare-ups correlated with SCS, AIS, and K6. Finally, medication effectiveness correlated with all pain-related measures except for HADS-A. Among these, the strongest correlation was observed between the cope with pain and HADS-D (r = 0.49, *p* < 0.01), while the weakest was observed between cope with pain and PSEQ (r = −0.34, *p* < 0.01).

## 4. Discussion

The MPIC is a novel questionnaire developed by Gagnon et al. to assess changes in pain-related measures through patients’ impressions following treatment. It can solely evaluate eight essential goals intended to be achieved via interdisciplinary pain management without a combination of additional specific questionnaires [3]. Furthermore, its capacity for retrospective assessment allows for the evaluation of treatment effects even in the absence of pretreatment data.

The findings of our study indicated significant improvements in all pain-related questionnaires except for the Depression Scale (HADS-D), suggesting that our interdisciplinary pain management approach is effective in the short term. Although this study contains several types of pain management, and did not focus on a specific treatment, the dataset with several improved scores supports the validity of the MPIC, which assesses multifaceted treatment effects. The MPIC domains consistently scored below 4, where 4 indicates no improvement and lower scores represent greater improvement, suggesting overall improved impressions of change across multiple pain dimensions.

A factor loading of the CFA, which was performed to evaluate the structural validity in this study, indicates how strongly an item represents the factor, with higher loadings reflecting items that are more crucial for evaluating the model. Given that a factor loading of 0.5 or higher is generally regarded as good [29], most factor loadings demonstrated moderate to strong associations with the single factor model of the MPIC. Manage pain flare-ups exhibited a comparatively lower factor loading (0.49), although it still contributed to the overall multidimensional structure of the scale. This lower factor loading may reflect a restricted range of scores, potentially indicating that participants had limited opportunities to improve their pain management skills in this area. Given the significant associations observed between the manage pain flare-ups and changes in several psychological measures, it is possible that this domain captures psychological aspects of pain management. Psychological therapy may have the potential to enhance pain management skills and improve scores within this domain. However, access to psychological therapy for chronic pain is limited in Japan due to the fact that it is not covered by the universal health insurance system. As a result, manage pain flare-ups seemed to exert a comparatively limited influence on the impression of clinical improvement.

Goodness-of-fit analysis indicated a marginal fit for the one-factor model of the MPIC. Although the RMSEA suggested poor fit, this may be attributed to the relatively small sample size in this study, as RMSEA tends to overestimate misfit in smaller samples [30]. Also, the limited number of items may have reduced degrees of freedom, explaining the poor RMSEA [30]. The CFI (0.86) did not meet the generally accepted threshold for good fit (>0.90) and is therefore best characterized as indicating marginal fit. Conversely, the SRMR (0.06), which is less influenced by sample size and more stable, was within the criteria of good fit (<0.08) indicating a good fit in this study. Taken together, these findings provide preliminary, rather than definitive, support for the structural validity of the MPIC.

The MPIC is intended for use within interdisciplinary pain centers. Given that these centers employ multifaceted treatment approaches to address complex chronic pain issues, it is reasonable to expect that the MPIC’s multiple domains would demonstrate a unified directionality. The data used in this study were collected from patients attending a pain center, which may explain the one-factor model as indicated by the EFA. In settings where treatment is focused on a specific aspect of pain management, variations between the MPIC domains might be more readily detectable. From this perspective, further research using data from diverse facilities would be beneficial. However, it is also recognized that comprehensive, multifaceted treatment approaches are generally more effective for complex, refractory chronic pain conditions. Therefore, it remains plausible that the MPIC will primarily function as a one-factor model.

The AVE reflects how well each item consistently represents an underlying construct. A higher AVE value implies that the items consistently represent the factor, demonstrating better convergent validity, suggesting the MPIC effectively encompasses diverse clinical profiles of patients with chronic pain. In the reliability analysis, Cronbach’s alpha was 0.93, indicating excellent internal consistency, which aligns with previous studies [3].

Furthermore, the ICC was 0.52, suggesting moderate test–retest reliability. A correlation coefficient of 0.70 or higher is generally considered a strong consistency between the two time points [31]. Although statistically significant correlations were identified with the domains of sleep, cope with pain, and manage pain flare-ups, their coefficients were below 0.70, suggesting potential instability. The low correlation coefficients would be based on the small sample size compared to the fluctuations in participants’ conditions. As a whole, most scores remained stable, with only slight changes, suggesting good reproducibility over time. While pain can fluctuate over time, causing variations in responses at different time points, the moderate test–retest reliability indicates that MPIC responses remain consistent across multiple tests. Thus, the MPIC is suitable for repeated use to effectively track treatment effects over time.

We investigated the external validity of the MPIC by examining the correlations between its domains and changes in pain-related measures (Figure 4). Several MPIC domains showed significant correlations with changes in pain intensity, functional disability, pain catastrophizing, self-efficacy, psychological distress, and sleep quality. Correlation of the pain domain with VAS and other clinical scales underscores its relevance for pain intensity assessment. Prior research has reported that poor sleep quality and insomnia increase the risk of chronic pain [21], and insomnia can amplify pain sensitivity [32]. The significant association between the sleep domain and the AIS, along with its correlation with the pain domain and VAS, further supports the usefulness of the MPIC.

Notably, change in the SCS demonstrated significant correlations with all MPIC domains, and PSEQ showed significant correlations with all except the sleep domain. Catastrophizing measured by the SCS is an important clinical feature in pain management because the pathological thoughts link to chronic pain and disability [33]. In addition, higher self-efficacy is associated with better physical function among individuals with chronic pain [34], resulting in improvement of the PSEQ, measuring self-efficacy in painful situations, correlated significantly to all MPIC domains except for sleep.

While pain, sleep, mood, and physical functioning are key components of quality of life and are commonly utilized indicators in numerous studies, a unique feature of the MPIC is additional domains, such as cope with pain, manage pain flare-ups, and medication effectiveness, which were specifically designed to assess outcomes related to chronic pain treatment. There are currently no standardized patient-reported measures that match each MPIC domain. Our findings suggest that improvements in these domains may be associated with less catastrophizing and better self-efficacy in patients with chronic pain. The MPIC is a straightforward and practical tool for regular pain management and may capture multidimensional changes in patients’ conditions, requiring just one post-treatment assessment. In summary, consistent with previous research, our findings indicate that the MPIC effectively reflects changes across multiple key domains of interdisciplinary pain management, encompassing both psychological and physical aspects [3]. The MPIC can serve as a comprehensive tool to evaluate not only pain intensity but also patients’ coping abilities and treatment effectiveness in interdisciplinary pain management.

This study acknowledges several limitations that warrant consideration when interpreting the findings. First, the small sample size, particularly the subgroup that completed the test–retest analysis (n = 20), may have limited the statistical power for robust test–retest reliability assessment. The sample size also precluded meaningful subgroup analyses based on pain location, age, or gender, factors that may influence psychometric performance. Although the sample size met minimal conventional guidelines for confirmatory factor analysis, larger samples are increasingly recommended for more robust model evaluation [35,36]. A larger sample would enhance the robustness and accuracy of the data, providing more reliable insights into the effectiveness of the MPIC. Second, as a single-center study conducted within our Interdisciplinary Pain Center, selection bias may be present, potentially limiting the generalizability of the results to broader populations or other patient groups, such as those with cancer pain. Third, the retrospective self-report nature of the MPIC introduces the potential for response-shift and recall bias, which could influence participant scores. Fourth, the inclusion of various pain types and treatments, along with the variability in follow-up length inherent in a routine clinical setting, prevented specific analyses related to particular pain conditions or treatment modalities. While acknowledging this heterogeneity, the findings suggest the MPIC’s potential utility as an evaluation tool across diverse pain and treatment contexts. Finally, the absence of well-established questionnaires directly measuring medication effectiveness beyond pain intensity presents a challenge. In clinical practice, improvements in medication effectiveness often involve reducing analgesic dosages, yet the relationship between medication effectiveness and pain outcomes remains under-investigated.

Future research should prioritize longitudinal studies employing larger, more heterogeneous samples to enhance the rigor and scope of findings. Such investigations could improve the robustness and generalizability of results while also allowing for exploration of potential enhancements to model fit. Multicenter study designs may further mitigate selection bias. Moreover, future research should examine the applicability of the MPIC across diverse pain conditions and treatment settings.

## 5. Conclusions

Our findings suggest that the Japanese version of the MPIC is a valid, reliable, brief, and retrospective instrument for multidimensional assessment in Japanese patients with chronic non-cancer pain, and is suitable for evaluating treatment outcomes in interdisciplinary pain management. Future multicenter use of the MPIC will support confirming and generalizing these findings.

## Figures and Tables

**Table 1:** Demographic characteristics (N = 101).

Age (years), mean (SD)	54.5 (14.6)
Women, n (%)	66 (65.3)
BMI (kg/m^2^), mean (SD)	21.7 (3.8)
Pain duration (month), median (min, max)	42 (1, 660)
Treatment period (days), median (min, max)	169 (28, 637)
Pain location, n (%)	
Upper limbs	57 (56.4)
Head and/or neck	52 (51.5)
Lower limbs	51 (50.5)
Chest and/or abdomen	25 (24.8)
Back	24 (23.8)
Perineal	3 (3.0)

SD: standard deviation, BMI: body mass index.

**Table 2:** Treatment outcome measures.

Mean (SD)	Pre	Post	*p* Value
VAS	59.9 (22.1)	52.8 (22.3)	0.003
PDQ	19.4 (6.5)	17.9 (6.3)	0.002
PDI	27.4 (12.0)	23.8 (12.0)	0.003
SCS	8.0 (3.6)	5.8 (3.5)	<0.001
PSEQ	22.6 (12.1)	26.9 (11.4)	<0.001
AIS	9.3 (4.7)	8.1 (4.5)	0.013
HADS-A ^#^	10.5 (5.3)	8.7 (3.9)	0.004
HADS-D ^#^	9.5 (4.3)	8.9 (4.6)	0.160
K6 ^$^	8.4 (6.2)	6.6 (5.4)	0.002
MPIC, mean (SD)			
Overall status		3.3 (1.3)	
Pain		3.4 (1.3)	
Sleep		3.7 (1.2)	
Mood		3.4 (1.3)	
Physical functioning		3.7 (1.3)	
Cope with pain		3.3 (1.3)	
Manage pain flare-ups		3.4 (0.9)	
Medication effectiveness		3.4 (1.0)	

Pre- and post-data were compared using a paired *t*-test. ^#^ HADS and ^$^ K6 were assessed in 35 and 66 participants, respectively. SD: standard deviation, VAS: Visual Analog Scale, PDQ: PainDETECT Questionnaire, PDI: five-item version of Pain Disability Index, SCS: Symptom Catastrophizing Scale, PSEQ: Pain Self-Efficacy Questionnaire, HADS-A: Hospital Anxiety and Depression Scale—anxiety, HADS-D: Hospital Anxiety and Depression Scale—depression, K6: Kessler Psychological Distress Scale-6 items, AIS: Athens Insomnia Scale, MPIC: Multidimensional Evaluation Scale for Patient Impression Change.

**Table 3:** Summary of the validity and reliability analyses.

Category	Index Name	Results	Criterion of Acceptance
Validity			
Construct Validity	RMSEA	0.23	<0.1 [22]
	CFI	0.86	>0.8 [23]
	SRMR	0.06	<0.08 [24]
Convergent validity	AVE	0.58	≥0.5 [25]
Reliability			
Internal consistency	Cronbach’s alpha	0.93	≥0.7 [26,27]
Test–retest reliability	ICC	0.52	>0.5 [28]

RMSEA: root mean square error of approximation, CFI: comparative fit index, SRMR: standardized root mean square residual, AVE: average variance extracted, ICC: intraclass correlation coefficient.

## Data Availability

The data presented in this study are available on request from the corresponding author due to ethical restrictions.
